# Short-term effects of air pollution: a panel study of blood markers in patients with chronic pulmonary disease

**DOI:** 10.1186/1743-8977-6-25

**Published:** 2009-09-26

**Authors:** Katharina Hildebrandt, Regina Rückerl, Wolfgang Koenig, Alexandra Schneider, Mike Pitz, Joachim Heinrich, Victor Marder, Mark Frampton, Günter Oberdörster, H Erich Wichmann, Annette Peters

**Affiliations:** 1 Ludwig-Maximilians-University of Munich, IBE Department of Epidemiology, Munich, Germany; 2 Helmholtz Zentrum München, German Research Center for Environmental Health, Institute of Epidemiology, Munich, Germany; 3 University of Ulm Medical Center, Department of Internal Medicine II, Cardiology, Ulm, Germany; 4 Environmental Science Centre, University of Augsburg, Germany; 5 Rochester School of Medicine and Dentistry, Department of Medicine and Dentistry - Vascular Medicine, Rochester, NY, USA; 6 Rochester School of Medicine and Dentistry, Department of Medicine and Dentistry - Pulmonary and Critical Care Unit, Rochester, NY, USA; 7 Department of Environmental Medicine, Rochester School of Medicine and Dentistry, Rochester, NY, USA; 8 Helmholtz Zentrum München, Focus-Network Aerosols and Health, Munich, Germany

## Abstract

**Background:**

Growing evidence indicates that ambient air pollution is associated with exacerbation of chronic diseases like chronic pulmonary disease. A prospective panel study was conducted to investigate short-term changes of blood markers of inflammation and coagulation in response to daily changes in air pollution in Erfurt, Germany. 12 clinical visits were scheduled and blood parameters were measured in 38 male patients with chronic pulmonary disease during winter 2001/2002. Additive mixed models with random patient intercept were applied, adjusting for trend, weekday, and meteorological parameters. Hourly data on ultrafine particles (UFP, 0.01-0.1 μm), accumulation mode particles (ACP, 0.1-1.0 μm), PM_10 _(particulate matter <10 μm in diameter), elemental (EC) and organic carbon (OC), gaseous pollutants (nitrogen monoxide [NO], nitrogen dioxide [NO_2_], carbon monoxide [CO], and sulphur dioxide [SO_2_]) were collected at a central monitoring site and meteorological data were received from an official network. For each person and visit the individual 24-hour average of pollutants immediately preceding the blood withdrawal (lag 0) up to day 5 (lag1-4) and 5-day running means were calculated.

**Results:**

Increased levels of fibrinogen were observed for an increase in one interquartile range of UFP, PM_10_, EC, OC, CO, and NO revealing the strongest effect for lag 3. E-selectin increased in association with ACP and PM_10 _with a delay of one day. The ACP effect was also seen with the 5-day-mean. The pattern found for D-dimer was inconsistent. Prothrombin fragment 1+2 decreased with lag 4 consistently for all particulate pollutants. Von Willebrand factor antigen (vWF) showed a consistent decrease in association with almost all air pollutants with all lags except for lag 0. No associations were found for C-reactive protein, soluble intercellular adhesion molecule 1, serum amyloid A and factor VII.

**Conclusion:**

These results suggest that elevated concentrations of air pollution are associated with changes in some blood markers of inflammation and coagulation in patients with chronic pulmonary disease. The clinical implications of these findings need further investigation.

## Background

Growing evidence indicates that air pollution is associated with disease exacerbation and increased risk of cardiovascular death in cardiovascular patients [1] but also in patients with chronic obstructive pulmonary disease (COPD) [2-4]. Hypothesised pathways between particulate air pollution and cardiovascular disease include a particle induced lung inflammation that affects the endothelium, thrombotic potential and fibrinolytic balance in ways that favour plaque rupture and thrombosis and a particle translocation in to the blood that directly affects the endothelium [5]. Associations between ambient air pollution and markers of inflammation and coagulation have been shown in patients with cardiovascular disease [6]. In addition, increased circulating levels of such mediators of inflammation and coagulation have been found in patients with COPD [7,8]. This study was designed specifically to investigate whether ambient air pollution leads to an enhanced systemic inflammatory response and changes in blood coagulability in patients with chronic pulmonary disease. Repeated measurements of blood markers were compared with concurrent levels of gaseous and particulate ambient air pollutants on a daily basis. Our hypothesis was that inflammation and coagulation markers would increase in association with higher levels of air pollution. The following parameters were measured: fibrinogen, C-reactive protein (CRP), serum amyloid-A (SAA), E-selectin, soluble intercellular adhesion molecule-1 (sICAM-1), von Willebrand factor antigen (vWF), prothrombin fragment 1+2, D-dimer, and factor VII (FVII).

## Methods

### Study design

A prospective panel study was performed between October 15, 2001 and May 6, 2002 in Erfurt, Germany. 38 male patients with chronic pulmonary disease were recruited by practitioners or through newspaper advertisement and invited for twelve clinical visits. In the repeated measurement design each patient serves as his own control and therefore baseline characteristics of each patient and other unknown confounders are automatically adjusted for. At each visit a short interview was conducted and blood samples were taken. At the first visit a baseline questionnaire was administered regarding health status, pulmonary and cardiac symptoms, medication use and smoking history.

Written informed consent was obtained from all participants and the protocol was approved by the Ethics Committee Bavaria, Germany.

All methods used in the study were conducted according to Standard Operating Procedures (SOP).

### Ambient air pollution measurements and exposure assessment

Ambient air pollutants were recorded hourly at a fixed monitoring station in the vicinity of the city centre of Erfurt representing urban background levels. The particle number size distribution was measured on a continuous basis using two instruments covering different size ranges. Both instruments, data analyses and quality control were described in detail by Pitz et al. [9]. Briefly, number concentration for ultrafine particles (UFP, 0.01-0.1 μm) and accumulation mode particles (ACP, 0.1-1.0 μm) were calculated from each spectrum. For the online measurement of the mass concentration of PM_10 _(particulate matter with an aerodynamic diameter smaller than 10 μm) we applied a Tapered Element Oscillating Microbalance (TEOM Model1400a, Thermo Fisher Scientific Inc., USA). Continuous mass concentration measurements of elemental (EC) and organic carbon (OC) were determined with an Ambient Carbon Particulate Monitor (ACPM, Model 5400, Thermo Fisher Scientific Inc., USA). Concurrent data on meteorological variables like temperature, relative humidity, and barometric pressure as well as gaseous pollutants like nitrogen monoxide (NO), nitrogen dioxide (NO_2_), carbon monoxide (CO), and sulphur dioxide (SO_2_) were obtained from the Thuringia state monitoring network station in Erfurt. Missing values for UFP, PM_10_, SO_2_, NO_2_, NO, temperature, and relative humidity were imputed by a linear regression model based on data from parallel measurements. The R-squares for the UFP regression model was 0.93 and for PM_10 _0.79.

For each subject and visit, individual 24-hour averages of pollutants immediately preceding the clinical visit (0-23 hours [lag 0], 24-47 hours [lag 1], 48-71 hours [lag 2], 72-95 hours [lag 3], 96-119 hours [lag 4]) as well as a 5 day average prior to the examination were calculated if more than 2/3 of the hourly measurements were available for this time period.

### Clinical measurements

For each patient 12 clinical visits were scheduled biweekly on the same weekday (Monday to Friday) and same time (8:00 am to 5:00 pm).

At each visit EDTA and citrate plasma samples were taken (Becton Dickinson, NJ, USA). Samples were centrifuged and aliquots were immediately stored at -20°C until analysis. CRP (high-sensitivity assay), SAA, and fibrinogen were analyzed by immunonephelometry (Dade Behring, Marburg, Germany). S-ICAM-1, E-selectin (R&D Systems, Wiesbaden, Germany), and prothrombin fragment 1+2 (Dade Behring, Marburg, Germany) were measured by means of a commercial ELISA. D-dimer and vWf were analyzed using an immuno turbidimetric method and FVII by clotting time measurement (Stago, France).

### Study population

Exclusion criteria were pacemaker, insulin dependent diabetes mellitus, recent myocardial infarction (MI), bypass-surgery or balloon dilatation less than three months ago, unstable angina, asbestosis and silicosis. Additionally, patients on anticoagulation therapy and those who were hospitalized more than three times in the six months prior to the start of the study because of their lung disease were not eligible. Moreover, patients who took antibiotics more than four times in the six months prior to the start of the study or the winter (October to March) prior to the study could not participate.

The data of 38 of 42 males could be included in the analyses. One patient was excluded because of cancer diagnosed after the study had started. Data of three other people could not be used as they had only one single measurement.

Out of 456 possible visits a total of 452 visits were accomplished, indicating completeness of over 99%. 21 blood samples (5% of total visits) were excluded from analyses because the patients reported fever during the last week. Thirty additional blood samples (7% of total visits) were withdrawn because clinical examiners diagnosed an acute respiratory infection. Furthermore, in 46 visits (10% of total visits) patients reported an acute respiratory infection during the two weeks prior to the examination without current signs of infection. For these cases, a risk variable (airway infection) was built and tested for confounding effects. Finally, not all patients were able to give the intended amount of blood at each visit. Therefore between 347 (77% of total visits) and 380 (84% of total visits) blood samples remained for analysis depending on the marker.

### Statistical analyses

The longitudinal data were analyzed using SAS Version 9.1 (SAS Institute Inc.) using additive mixed models with random patient intercepts. To model the correlation between repeated measurements in each patient we assumed a compound symmetry structure for the covariance matrix, because the half-lives of the markers were shorter than the intervals between visits.

Prothrombin fragment 1+2, CRP, and D-dimer were log transformed because their residuals remained skewed after multivariate modelling.

In a first step, a confounder selection without blood markers was conducted for every blood marker separately. Model fit was based on the lowest value of the Akaike Information Criterion (AIC). Long-term time trend and air temperature, relative humidity, and barometric pressure, each with lag 0 to lag 3, were considered as potential confounders. In addition, five risk variables (airway infection, medical attendance, hospital admission, and corticosteroid or antibiotic intake during the two weeks prior to the visit) were created and included into the model if this improved the model fit. Also, an indicator variable for weekday of the visit was added to the final confounder model. Penalised splines (P-splines) were used to allow for non-linear confounder adjustment.

Exposure variables were added one by one to the final model as linear terms. Effect estimates are presented as percent change of the arithmetic mean or geometric mean (after log transformation) together with 95% confidence intervals (CI) based on an increase in air pollution concentration from the 1^st ^to the 3^rd ^quartile (interquartile range, IQR).

We performed a sensitivity analysis for all blood markers with selected air pollutants excluding the seven current smokers. Also, subgroup analyses for asthmatics and non-asthmatics were conducted.

## Results

### Patient characteristics

Patient characteristics are shown in table 1. The study population consisted of 38 men, aged 34 to 78 years. Seven patients were current smokers and most of the remaining participants were ex-smokers. The majority of ex-smokers had stopped smoking more than ten years before the start of the study. Two participants had stopped three years and four years before the start of the study, respectively, and two participants had stopped one year before the start of the study. Only current smokers reported smoking in the past 24 hours before the visits while none of the non- or ex-smokers had smoked before any of the visits.

**Table 1:** Characteristics of the study population, 38 men with a history of chronic pulmonary disease in Erfurt, Germany, winter 2001/2002

**Variable**			**Mean ± DS^†^**
**Age [years]**			53.8 ± 12.3
BMI* [kg/m^2^]			25.5 ± 3.7
			**N (%)**

**History of**	COPD		
	I (mild)	FEV_1_/FVC < 70% and FEV_1_/FEV_1 predicted _> 80%	2 (5)
	II (moderate)	FEV_1_/FVC < 70% and 50% ≤ FEV_1_/FEV_1 predicted _< 80%	7 (18)
	III (severe)	FEV_1_/FVC < 70% and 30% ≤ FEV_1_/FEV_1 predicted _< 50%	5 (13)
	Chronic bronchitis^†^		29 (76)
	Emphysema^†^		5 (13)
	Bronchial asthma^†^		20 (53)
	Coronary heart disease^†^		11 (29)
	Angina pectoris^†^		15 (40)
	Myocardial infarction^†^		8 (21)
	Bypass surgery/balloon dilatation^†^		4 (11)
	Hypertension^†^		14 (37)
	Chronic heart failure^†^		2 (5)
	Cardiac arrest^†^		1 (3)
	Diabetes mellitus Type 2^†^		2 (5)
	Allergic rhinitis^†^		11 (29)
	Sleep apnea^†^		4 (11)
	Chronic kidney disease^†^		2 (5)
**Smoking history**	Ex smoker		23 (61)
	Current smoker		7 (18)

**Long-term medication use**	Patients with long-term medication		35 (92)
	β_2_-agonists		22 (58)
	Theophylline		17 (45)
	Glucocorticosteroids		20 (53)
	Antibiotics		5 (13)
	Statins		6 (16)

COPD, Chronic obstructive pulmonary disease; ^‡ ^SD, standard deviation; * BMI, Body mass index; ^†^"ever physician diagnosed" determined by an interview with trained nurses; ^# ^FEV_1_, forced expiratory volume in 1 second; ^§ ^FVC, forced vital capacity

### Blood parameters

Levels of blood parameters are summarized in table 2. Spearman correlations were found between the acute phase response parameters (CRP and fibrinogen, r = 0.64; CRP and SAA, r = 0.43; SAA and fibrinogen, r = 0.39) and a moderate correlation was found between the adhesion molecules E-selectin and sICAM-1 (r = 0.37). Markers of the acute phase response were not correlated with adhesion molecules (r = -0.13 to 0.29). Correlation was also poor between the coagulation markers (r = -0.01 to 0.35).

**Table 2:** Biomarkers for 38 male Patients with history of chronic pulmonary disease in Erfurt, Germany, between October 2001 and May 2002

**Variable**	**No.**	**Mean**	SD*	**Min.**	**Med.**	**90%**	**Max.**
**Fibrinogen** [g/l]	380	3.1	0.7	0.9	2.9	4.1	6.3
**SAA** [mg/l]^†^	380	7.7	44.0	0.8	2.9	6.8	718.0
**CRP** [mg/l]^‡^	380	3.8	8.4	0.2	1.6	7.5	127.0
**E-selectin** [ng/ml]	380	50.3	20.9	4.0	47.3	77.7	116.2
**sICAM-1** [ng/ml]^§^	380	302.8	104.3	120.7	274.4	419.0	855.9
**Faktor VII** [% activity]	349	131.2	35.3	53.0	125.0	180.0	315.0
**vWF** [% activity]^#^	349	126.4	43.4	25.0	126.0	186.0	213.0
**D-dimer** [μg/ml]	349	0.5	0.6	0.02	0.3	0.9	3.8
**Prothrombin fragment 1+2** [nmol/l]	347	2.2	1.0	0.6	2.1	3.5	6.0

* SD, standard deviation; ^† ^SAA, serum amyloid A; ^‡ ^CRP, C-reactive protein; ^§ ^sICAM-1, soluble inter-cellular adhesion molecule 1; ^# ^vWF, von Willebrand factor antigen

### Air pollutants

**Figure 1:**
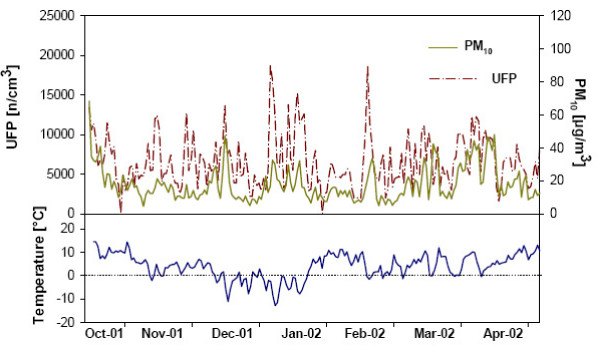
**Time series of number concentrations of particles sized 0.01 to 0.1 μm (ultra fine particles, UFP) and mass concentrations of particles less than 10 μm in diameter (PM_10_) with air temperature in Erfurt, Germany between October 2001 and May 2002**.

The descriptive statistics of daily average concentrations of particulate, gaseous, and meteorological parameters are presented in table 3. For the UFP measurements, 1257 missing values from the differential mobility particle sizer (DMPS) were replaced by a combination of condensation particle counter (CPC) and scanning mobility particle sizer (SMPS) measurements. 26 missing PM_10 _data were replaced by measurements from three other sites. Regarding temperature and relative humidity, only three measurements were missing and obtained from a different measurement site. 156 NO_2 _measurements and 280 NO measurements were substituted by data from other measurement sites. As the exposure variables are missing independent of the health outcomes they are assumed to be missing completely at random (MCAR) which will not bias the associations. Figure 1 illustrates UFP, PM_10_, and temperature during the course of the study period. Spearman correlation coefficients were calculated for different air pollution measurements. A high correlation was found between ACP and PM_10 _(r = 0.9) as well as between OC and EC (r = 0.93). UFP were moderately correlated to ACP (r = 0.75). Correlations between particles and gaseous pollutants were highest between ACP and CO (r = 0.72) and NO_2 _(0.73), respectively.

**Table 3:** Descriptive statistics of daily average concentrations of ambient particulate and gaseous air pollutants and meteorological variables in Erfurt, Germany, between October 2001 and May 2002

**Variable**	**No.**	**Mean (± SD^§^)**	**Min.**	**Median**	**Max.**	**IQR^#^**	**IQR^5#^**
**Number concentration [n/cm^3^]**							
UFP*	207	6604 (± 3147)	1547	5972	18845	3827	2918
ACP*	146	1094 (± 773)	211	866	3886	907.3	914.2
**Mass concentration [μg/m^3^]**							
PM_10 _*	208	17.73 (± 10.29)	4.38	15.03	57.27	11.43	9.91
OC*	208	1.04 (± 0.46)	0.43	1.04	2.87	0.52	0.37
EC*	208	2.60 (± 1.96)	0.40	1.97	10.63	1.98	1.76
SO_2 _*	206	3.54 (± 2.61)	3.00	3.00	37.22	0.17	0.41
NO_2 _*	208	25.37 (± 9.90)	6.54	24.94	55.74	12.94	9.70
CO*	208	0.42 (± 0.16)	0.14	0.39	1.07	0.17	0.14
NO*	199	13.32 (± 10.48)	4.00	9.63	56.43	11.30	10.04
**Meteorology**							
**Air temperature** [°C]	208	4.23 (± 5.49)	-12.83	4.79	15.71	8.13	6.85
**Barometric pressure **[hPa]	208	979.6 (± 10.5)	952.8	979.5	1002.3	14.6	11.8
**Relative humidity** [%]	208	82.22(± 11.72)	48.75	84.40	100.00	15.44	12.27

* UFP, ultrafine particles, number concentration of particles with a size range of 0.01 to 0.1 μm in diameter; ACP, accumulation mode particles, particles with a size range of 0.1 to 1.0 μm; PM_10_, mass concentration of particles less than 10 μm in diameter; OC, organic carbon; EC, elemental carbon; SO_2_, sulfur dioxide; NO_2_, nitrogen dioxide; CO, carbon monoxide; NO, nitrogen monoxide; ^§ ^SD, standard deviation; ^# ^IQR, interquartile range; IQR^5^, Interquartile range for 5 day average

### Regression results

Results for the linear regression of different blood markers are summarized in table 4 and table 5.

**Table 4:** Effects of particulate pollutants on fibrinogen, E-selectin, and vWF presented as % change from the arithmetic mean in the blood marker (linear regression) per increase in IQR^† ^air pollutant; 38 men with chronic pulmonary disease in Erfurt, Germany, between October 2001 and May 2002

**Polutant**	**Fibrinogen**		**E-Selectin**		**vWF^††^**	
	**%change AM^††^**	**95%CI**	**%change ^AM††^**	**95%CI**	**%change AM^††^**	**95%CI**

**UFP**						
Lag0#	0.5	-1.8 ; 2.9	-1.2	-3.9 ; 1.4	0.1	-2.7 ; 2.9
Lag1#	2.5 *	0.2 ; 4.9	0.2	-2.7 ; 3.1	-0.4	-3.3 ; 2.5
Lag2#	-1.6	-4.3 ; 1.0	-2.0	-5.1 ; 1.0	-4.1 **	-7.3 ; -1.0
Lag3#	3.3 **	1.0 ; 5.6	-1.1	-4.3 ; 2.0	-4.3 **	-6.9 ; -1.6
Lag4#	1.5	-0.5 ; 3.5	1.6	-1.1 ; 4.2	-4.0 **	-6.4 ; -1.6
5-day-mean#	3.1 *	0.2 ; 6.0	-0.9	-4.5 ; 2.7	-6.1 ***	-9.7 ; -2.6
**ACP**						
Lag0#	0.2	-3.7 ; 4.1	3.1 *	0.2 ; 6.1	2.6	-2.6 ; 7.7
Lag1#	0.5	-2.7 ; 3.7	4.0 **	1.1 ; 6.9	-2.3	-6.4 ; 1.7
Lag2#	-1.0	-3.9 ; 1.8	2.9 **	0.4 ; 5.3	-4.9 **	-8.1 ; -1.6
Lag3#	1.3	-1.1 ; 3.7	1.3	-1.0 ; 3.6	-4.2 **	-7.2 ; -1.2
Lag4#	0.8	-1.7 ; 3.3	2.1 ^†^	0.0 ; 4.2	-6.3 ***	-9.7 ; -2.8
5-day-mean#	0.5	-3.6 ; 4.6	5.4 **	1.7 ; 9.1	-6.8 *	-12.2 ; -1.5
**PM10**						
Lag0#	0.5	-1.7 ; 2.7	-1.1	-3.8 ; 1.6	2.1	-0.8 ; 5.0
Lag1#	0.1	-1.8 ; 2.0	2.6 *	0.2 ; 5.1	-1.6	-3.9 ; 0.7
Lag2#	-0.5	-2.3 ; 1.3	1.1	-1.1 ; 3.4	-2.2 *	-4.3 ; -0.1
Lag3#	2.4 **	0.6 ; 4.1	-0.1	-2.5 ; 2.3	-2.4 *	-4.7 ; -0.2
Lag4#	1.8 *	0.1 ; 3.5	0.3	-2.0 ; 2.6	-1.9	-4.2 ; 0.5
5-day-mean#	1.9	-0.5 ; 4.3	1.4	-1.9 ; 4.7	-3.3 *	-6.6 ; -0.1
**OC**						
Lag0#	0.1	-2.6 ; 2.7	-1.0	-4.1 ; 2.1	-1.1	-4.7 ; 2.4
Lag1#	0.1	-1.9 ; 2.1	2.7 *	0.2 ; 5.1	-3.3 **	-5.7 ; -0.9
Lag2#	0.1	-1.7 ; 1.9	0.6	-1.6 ; 2.9	-4.6 ***	-6.7 ; -2.4
Lag3#	2.1 *	0.5 ; 3.6	0.1	-2.1 ; 2.2	-4.7 ***	-6.9 ; -2.6
Lag4#	1.5 ^†^	-0.1 ; 3.1	0.6	-1.5 ; 2.7	-4.5 ***	-7.0 ; -2.0
5-day-mean#	1.4	-0.5 ; 3.2	0.9	-1.5 ; 3.4	-6.4 ***	-9.1 ; -3.6
**EC**						
Lag0#	0.5	-1.9 ; 2.9	-2.1	-4.9 ; 0.7	-0.5	-3.6 ; 2.7
Lag1#	0.2	-1.7 ; 2.1	2.5 *	0.2 ; 4.7	-2.7 *	-4.9 ; -0.4
Lag2#	-0.5	-2.2 ; 1.3	0.8	-1.3 ; 2.9	-4.0 ***	-6.1 ; -1.9
Lag3#	2.5 **	0.9 ; 4.0	-0.2	-2.3 ; 1.9	-3.6 ***	-5.5 ; -1.7
Lag4#	1.8 *	0.3 ; 3.2	0.7	-1.2 ; 2.6	-3.3 ***	-5.4 ; -1.2
5-day-mean#	2.0 ^†^	-0.1 ; 4.1	0.9	-1.9 ; 3.7	-6.4 ***	-9.4 ; -3.4

^† ^IQR, interquartile range; ^††^AM: arithmetic mean; %-change: effect estimate obtained from regression divided by arithmetic mean of fibrinogen

vWF, von Willebrand factor antigen ^§ ^UFP, ultrafine particles number concentration of particles with a size range of 0.01 to 0.1 μm in diameter; ACP, accumulation mode particles, particles with a size range of 0.1 to 1.0 μm; PM_10_, mass concentration of particles less than 10 μm in diameter; OC, organic carbon; EC, elemental carbon

^# ^lag 0 (lag 1/lag 2/lag 3/lag 4/5-day-mean) = average pollutant concentration 0-23 (24-47/48-71/72-95/96-119/0-119) hours prior to blood withdrawal

* p < 0.05; ** p < 0.01; *** p < 0.001; ^† ^p ≤ 0.1

**Table 5:** Effects of air pollution on D-dimer and prothrombin fragment 1+2 presented as % change from the geometric mean in the blood marker (linear regression) per increase in IQR^† ^air pollutant; 38 men with chronic pulmonary disease in Erfurt, Germany, between October 2001 and May 2002

**Pollutant**	**D-Dimer**		**Prothrombin fragment 1+2**	
	**%change GM^††^**	**95%CI**	**%change GM^††^**	**95%CI**

**UFP**				
Lag0#	0.2	-8.0 ; 9.1	0.7	-3.5 ; 5.1
Lag1#	-8.8 ^†^	-17.3 ; 0.6	4.1 ^†^	-0.4 ; 8.8
Lag2#	-0.3	-8.8 ; 9.0	0.9	-3.5 ; 5.5
Lag3#	2.6	-5.6 ; 11.5	0.6	-3.3 ; 4.7
Lag4#	0.9	-6.4 ; 8.8	-2.6	-6.2 ; 1.2
5-day-mean#	-1.1	-10.6 ; 9.5	1.4	-4.4 ; 7.6
**ACP**				
Lag0#	-13.3 *	-24.0 ; -1.2	-0.9	-7.5 ; 6.2
Lag1#	-5.8	-16.8 ; 6.5	3.1	-2.5 ; 9.0
Lag2#	2.5	-7.0 ; 12.9	3.0	-1.5 ; 7.8
Lag3#	6.4	-2.6 ; 16.4	-1.2	-5.3 ; 3.0
Lag4#	6.2	-4.0 ; 17.5	-6.3 **	-10.5 ; -1.9
5-day-mean#	0.4	-14.0 ; 17.3	-1.1	-8.5 ; 7.0
**PM_10_**				
Lag0#	-10.3 **	-17.3 ; -2.7	0.7	-3.8 ; 5.5
Lag1#	-9.3 *	-16.0 ; -2.1	0.6	-2.9 ; 4.2
Lag2#	-1.7	-8.0 ; 5.1	0.6	-2.6 ; 3.8
Lag3#	4.3	-2.5 ; 11.6	-2.5	-5.9 ; 0.9
Lag4#	4.6	-2.4 ; 12.0	-5.1 **	-8.5 ; -1.6
5-day-mean#	-3.6	-12.4 ; 6.1	-3.5	-8.9 ; 2.1
**OC**				
Lag0#	-6.0	-14.2 ; 3.0	-0.9	-6.3 ; 4.9
Lag1#	-2.4	-9.5 ; 5.3	0.9	-2.8 ; 4.7
Lag2#	4.6	-1.9 ; 11.6	0.6	-2.9 ; 4.2
Lag3#	7.2 *	1.1 ; 13.7	-2.1	-5.7 ; 1.6
Lag4#	5.5 ^†^	-1.0 ; 12.5	-4.5 *	-8.3 ; -0.6
5-day-mean#	3.9	-3.0 ; 11.4	-2.2	-6.9 ; 2.8
**EC**				
Lag0#	-4.1	-11.9 ; 4.3	2.6	-2.5 ; 7.8
Lag1#	-3.5	-10.0 ; 3.5	1.7	-1.8 ; 5.3
Lag2#	3.2	-2.6 ; 9.4	1.2	-2.0 ; 4.6
Lag3#	5.3 ^†^	-0.3 ; 11.1	-2.5	-5.6 ; 0.8
Lag4#	3.8	-2.0 ; 9.9	-4.2 *	-7.3 ; -0.9
5-day-mean#	3.1	-4.3 ; 11.2	-1.6	-6.8 ; 3.9

^† ^IQR, interquartile range; GM: geometric mean (antilog of arithmetic mean of log-transformed variable); %-change: antilog of effect estimate obtained from regression minus 1 *100

^§ ^UFP, ultrafine particles number concentration of particles with a size range of 0.01 to 0.1 μm in diameter; ACP, accumulation mode particles, particles with a size range of 0.1 to 1.0 μm; PM_10_, mass concentration of particles less than 10 μm in diameter; OC, organic carbon; EC, elemental carbon

^# ^lag 0 (lag 1/lag 2/lag 3/lag 4/5-day-mean) = average pollutant concentration 0-23 (24-47/48-71/72-95/96-119/0-119) hours prior to blood withdrawal

* p < 0.05; ** p < 0.01; ^† ^p ≤ 0.1

### Inflammatory markers

**Figure 2:**
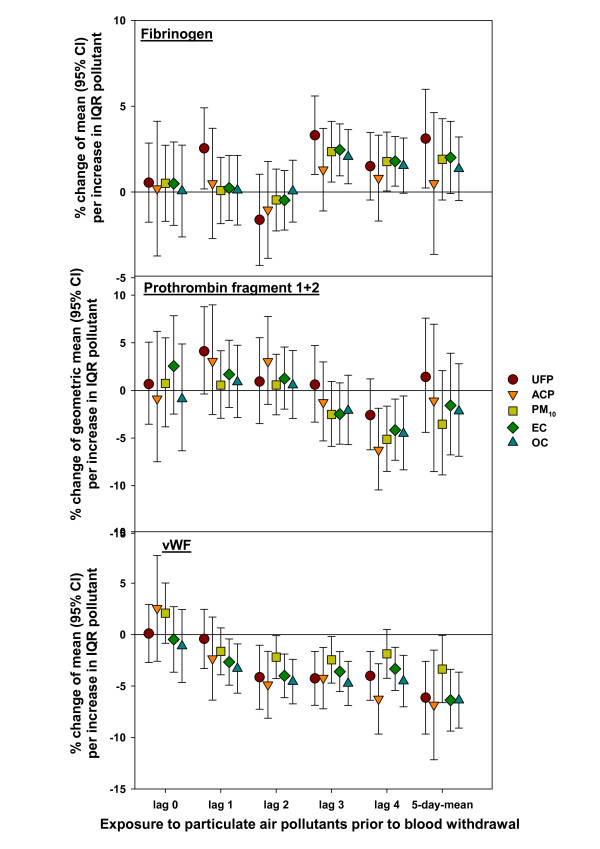
**Effects of particulate ambient air pollutants on fibrinogen, prothrombin fragment 1+2, and von Willebrand factor antigen (vWF), lag 0 to lag 4 and 5-day-mean exposure**. 38 men with chronic pulmonary disease in Erfurt, Germany, between October 2001 and May 2002. IQR, interquartile range; UFP, ultrafine particles number concentration of particles with a size range of 0.01 to 0.1 μm in diameter; ACP, accumulation mode particles, particles with a size range of 0.1 to 1.0 μm; PM_10_, mass concentration of particles less than 10 μm in diameter; OC, organic carbon; EC, elemental carbon; lag 0 (1, 2, 3, 4, 5-day-mean) = average pollutant concentration 0-23 (24-47; 48-71, 72-95, 96-119, 0-119) hours prior to blood withdrawal.

A consistent increase in fibrinogen was observed for lag 3 with all particulate pollutants except for ACP (table 4 and figure 2) and also with CO and NO (data not shown). For EC, CO, and NO this increase was also seen for lag 4 (data for CO and NO not shown). Additionally, the 5-day-mean concentrations of UFP demonstrated a positive significant association.

For E-selectin, a significant increase was found with ACP and PM_10 _for lag 1 (table 4). The ACP effect was also seen for lags 0 and 2 and for the 5-day-mean.

CRP, sICAM-1, and SAA showed no significant association with air pollutants (data not shown).

### Coagulation markers

An inconsistent pattern was determined for D-dimer with decreases for lag 0 (ACP and PM_10_) and lag 1 (UFP, PM_10_) and increases for lag 3 (OC, EC only borderline). This pattern was restricted to particulate pollutants (table 5).

Prothrombin fragment 1+2 decreased with lag 4 consistently with all particulate pollutants except for UFP (table 5 and figure 2) and with NO (data not shown).

A consistent decrease in vWF was ascertained for all particulate pollutants for lag 2 and for the 5-day-mean (table 5 and figure 2). Except for PM_10_, this decrease was also seen for lag 3 and lag 4. This pattern was also determined for the gaseous pollutants NO_2 _and CO (data not shown), however not that clear. A significant decrease of vWF was also found for lag 1 with EC and OC.

No significant associations between FVII and air pollutants were observed (data not shown).

### Sensitivity analyses

**Figure 3:**
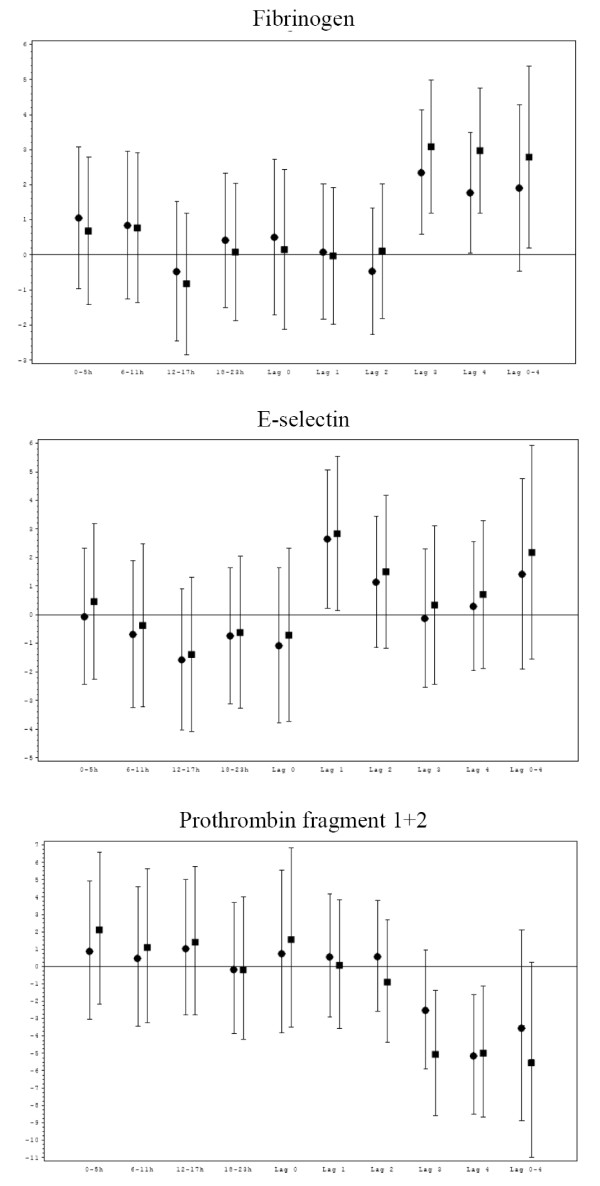
**Comparison of associations between blood markers and PM_10 _for all observations (black circle) and observations without smokers (black square) for fibrinogen, E-selectin and prothrombin fragment 1+2 lag 0 to lag 4 and 5-day-mean exposure**. 31 male non-smokers and 7 male smokers with chronic pulmonary disease in Erfurt, Germany, between October 2001 and May 2002. IQR, interquartile range; PM_10_, mass concentration of particles less than 10 μm in diameter; lag 0 (1, 2, 3, 4, 5-day-mean) = average pollutant concentration 0-23 (24-47; 48-71, 72-95, 96-119, 0-119) hours prior to blood withdrawal.

Excluding the seven smokers from the analyses hardly changed the results for most of the blood markers. Slightly stronger associations were seen for fibrinogen and prothrombin fragment 1+2 with PM_10_, EC and OC. Most other results remained stable, however with slightly wider confidence intervals, probably due to reduced number of observations (Figure 3).

Subgroup analyses showed that there were hardly any differences between asthmatics and non-asthmatics. Associations seemed slightly stronger for fibrinogen with UFP at lags 2 and 3 and D-Dimer for ACP with lag 1. For E-selectin few selected lags showed a slightly stronger association for non-asthmatics (data not shown).

## Discussion

### Summary

We examined the association between ambient air pollution and a range of blood markers of inflammation and coagulation in a panel of patients with chronic pulmonary disease. We hypothesised that blood markers would increase with increased levels of air pollution, confirming the hypothesis that particles cause lung inflammation, which might eventually induce a systemic inflammation.

We found a positive association between particulate air pollution and fibrinogen for lag 3 and with E-selectin especially for lag 1. Furthermore, vWf showed a consistent decrease in association with almost all measured pollutants. A negative association was also observed for lag 4 for prothrombin fragment 1+2. No clear pattern was found for D-dimer. No association between ambient air particles and most markers of inflammation like CRP, SAA, sICAM-1 or FVII were seen. Asthmatics do not seem to differ significantly from non-asthmatics.

### Inflammatory markers

Fibrinogen, an acute phase reactant and coagulation factor, is synthesised by hepatocytes and released in large amounts into the circulation in response to interleukin-6 (IL-6) stimulation [10]. Increased concentrations of fibrinogen are considered as indicators for an imbalance in the hemostatic system and as risk factors for arterial occlusive disorders such as MI or stroke [11]. During periods of COPD exacerbation, higher plasma levels of fibrinogen were found and hypothesised to be associated with cardiovascular morbidity and mortality [12]. Danesh et al. recently published a meta-analysis of 31 prospective studies which clearly showed a strong and independent association between elevated plasma levels of fibrinogen and various cardiovascular endpoints and total mortality [13]. This relationship suggests one explanation for the proven link between cardiovascular risk and obstructive lung disease [14].

We found small but consistently increased mean levels of fibrinogen in association with air pollution revealing the strongest effect for lag 3. Higher levels of fibrinogen in relation to PM_10 _were also measured in a sample of the U.S. population [15]. Additionally, similar results were found by Pekkanen et al. who reported higher fibrinogen levels in association with air pollution [16]. One year prior to this study a similarly designed study was conducted in patients suffering from coronary heart disease (CHD) [6]. The authors reported increases of CRP, SAA, and sICAM-1 in association with air pollution. These effects were restricted to higher values of these markers above the 90^th ^percentile using logistic regression models while linear regression analyses failed to reveal significant results. No association was found between fibrinogen and air pollution in these data. In our study, a logistic regression with a cutoff above the 90^th ^percentile was not possible because of poor intra-individual variability. The reduced variability may be due to the chronic inflammation associated with chronic pulmonary disease and elevated levels may indicate the severity of the disease. Furthermore, particulate and gaseous ambient air pollution markers tended to decrease from winter 2000/2001 to winter 2001/2002 in Erfurt while meteorological markers were comparable. This could be caused for example by a changed wind direction. It might also be possible that this is the result of reduced-emission road traffic or less factory fumes. However, the exposure to air pollution in our study population was lower and adverse effects might be smaller. This assumption is in agreement with Silkoff et al. [17] who observed detrimental associations of air pollution and worsened lung function in patients with COPD only for the second of two investigated winters with higher pollution levels.

In the UK, higher levels of CRP, the classical acute phase protein, were documented in elderly subjects in association to PM_10 _with a 3-day lag [18]. Moreover, the authors reported no significant effect on the acute phase reactant IL-6 levels but they noticed a reduction of fibrinogen in contrast to our results. Pope et al. also found positive associations between CRP and PM_2.5 _in elderly subjects in Utah [19]. Interestingly, this association disappeared after excluding the most influential subject determined by residual analysis. Furthermore, the MONICA-Augsburg study reported evidence of increased CRP for concentrations above the 90^th ^percentile in relation to air pollution in healthy men [20].

Besides, in patients with COPD some investigators failed to show a relationship between systemic and airway inflammation, suggesting different regulation of inflammation in the pulmonary and systemic compartment [21]. This might be one reason why we were not able to discover changes in plasma CRP, SAA, and sICAM-1. It is possible that analyses of sputum samples would have shown effects of air pollutants in our patient sample. Of interest, in this context, is the observation that in the case of COPD exacerbation, systemic inflammatory response is related to the magnitude of the lower rather than upper airway inflammation [22]. This leads to the assumption that particles with less diameter like UFP might be more harmful than larger particles because of a higher pulmonary deposition [23]. UFP have only been rarely assessed in the studies referenced above [6].

Our results support the idea that almost all examined particulate parameters and also some gaseous air pollutants are associated with increased fibrinogen levels in patients with chronic pulmonary disease. High fibrinogen levels are considered to be associated with a faster decline in lung function [24]. Therefore, air pollution might lead to a more rapid lung function decline in these susceptible patients as shown in the SAPALDIA study [25].

In addition, we observed increased levels of E-selectin that may reflect higher endothelial cell activation, as an increased expression of adhesion molecules is known to reflect activation of the vascular endothelium [26]. However, we did not find air pollution effects for the adhesion molecule sICAM-1.

### Coagulation markers

Patients with COPD are at higher risk of thrombotic events and increased levels of blood markers of coagulation have been found [7]. Prothrombin fragment 1+2 is cleaved from prothrombin when activated to thrombin, representing a marker of activation of the coagulation pathway [27]. Elevated levels have been found in COPD patients [7]. We observed decreases of prothrombin fragment 1+2 associated with increases of air pollution. This finding contrasts our hypothesis of enhanced coagulability. For most of the coagulation factors, changing the level between 50% and 150% has little effect on thrombin generation. But in the case of prothrombin, the rate of thrombin generation, peak activity reached and total amount of thrombin produced are proportional to the prothrombin level [28]. Thus, our results may reflect a delayed reduction of coagulation due to air pollutants.

We found similar results with vWF. On the one hand, vWF is known as a factor of the coagulation process [29] and on the other hand, vWF may serve as a marker of endothelial perturbation [30]. VWF is synthesized and stored in platelets, megacaryocytes, and endothelial cells [31]. Plasma concentrations of vWF are elevated in coronary artery disease [32] and may predict future cardiovascular mortality [33]. The results of vWF do not confirm our hypothesis that air pollution leads to enhanced endothelial cell activation and increased coagulability in patients with chronic pulmonary disease. However, vWF is a nonspecific marker [30] and cautious interpretation of our results is needed; especially as E-selectin showed quite a different pattern. D-dimer is a breakdown product of cross-linked fibrin, [34] and there is growing evidence that there may be an association between elevated levels of D-dimer and increased risk of future MI [35]. In our study, D-dimer showed an inconsistent pattern in association with air pollution parameters. It is possible that the observed increase found with lag 3 for EC and OC corresponds to the changes in fibrinogen. But previous studies report only modest correlations between D-dimer and fibrinogen [35], and we did not find high correlations either. The decrease of D-dimer found for lag 0 and lag 1 did not reflect the pattern of the other investigated markers of coagulability.

### Strengths and limitations

Our longitudinal study achieved over 99% of all scheduled visits indicating high compliance. The exposure to a variety of particles and gaseous pollutants was ascertained and numerous markers reflecting different pathways were measured. The detected effects are small and most probably only relevant to those subjects whose levels are at ends of the distribution.

We excluded blood samples from patients with obvious clinical signs of airway infection and adjusted for risk variables like airway infection, medical attendance, and hospital admission during the two weeks prior to the visit. This conservative approach may reflect another reason for the failure of verifying immunological effects for all markers in this relatively small study group.

Moreover, we adjusted for meteorological variables and long term time trend to minimize the possibility that the detected associations resulted from meteorological influences or seasonal differences. The co-morbidities of our subjects might also modify the effects of our results, as patients included in the study had different concomitant diseases and used several medications. Therefore, adjustments for an intake of corticosteroids and antibiotics were made.

The detected associations are only small and might be considered sub-clinical. However, even small changes may indicate a disrupted haemostasis. Moreover, small but consistent associations can give ideas for possible underlying pathways and are therefore interesting for research, even if they might be considered irrelevant in a clinical context. Finally, many acute little changes may also lead to atherosclerotic progression and chronic effects.

No assessments of indoor and individual personal exposures were conducted. However, Cyrys et al. [36] have shown that ambient air pollution can be used as surrogate measures for indoor exposure of for example PM_2.5 _and black smoke if major indoor sources are absent. Additionally, analyses in two European cities showed that ambient, indoor and personal concentrations of PM_2.5 _were highly correlated [37]. Ebelt et al. [38] also demonstrated that the component of personal exposure due to outdoor particles and ambient concentrations were highly correlated, especially for PM_2.5 _but also for PM_10 _and therefore data of ambient monitoring sites can be used in time series analyses.

Only about one third of our study population had a severe COPD and therefore comparison to some of the cited studies might be difficult. However, all of our patients were diagnosed with chronic pulmonary disease. Due to the selection criteria the results of this study are not representative of the general population but limited to male persons of similar age and disease status.

## Conclusion

This study provides evidence that ambient air pollution is associated with blood markers of inflammation and coagulation in patients with chronic pulmonary disease. These markers are known as risk factors for adverse cardiovascular events such as myocardial infarction and stroke and confirm therefore a hypothesised pathway between air pollution and cardiovascular disease in a panel of patients with chronic pulmonary disease. To what extend the observed changes may lead to disease exacerbation needs further investigation.

## Competing interests

The authors declare that they have no competing interests.

## Authors' contributions

KH performed the statistical analyses and drafted the manuscript; RR worked on the acquisition of the data, was substantially involved in the analyses and interpretation of the data and revised the manuscript critically; WK performed the analyses of some of the blood markers and reviewed the manuscript critically; AS was involved in the analyses and interpretation of the data and revised the manuscript critically; MP was involved in the acquisition of the air pollution data and reviewed the manuscript critically; JH was involved in the acquisition of the air pollution data and reviewed the manuscript critically; VM performed the analyses of some of the blood markers and reviewed the manuscript critically; MF performed the analyses of some of the blood markers and reviewed the manuscript critically; GO made substantial contribution to the design of the study and reviewed the manuscript critically; HEW was substantially involved in the design of the study and reviewed the manuscript critically; AP was substantially involved in the design of the study, the data acquisition and the interpretation of the results and reviewed the manuscript critically. All authors read and approved the final manuscript.
